# Adaptation of e-health impact questionnaire into Turkish: a validity and reliability study

**DOI:** 10.3389/fdgth.2025.1538475

**Published:** 2025-09-30

**Authors:** Mustafa Kafes, Serife Didem Kaya

**Affiliations:** Health Management Department, Necmettin Erbakan University, Meram, Türkiye

**Keywords:** Turkish, e-Health, questionnaire adaptation, digital health access, scale validation, cultural adaptation

## Abstract

**Introduction:**

e-Health refers to the use of information and communication technologies (ICT) for health-related services. e-Health aims to distribute scarce resources to areas in need more efficiently and to reduce the cost of health care delivery. However, although the health information on the web-based internet is sometimes provided by specialist and similar health professionals, it should be stated that the opposite can also happen. In this study, it is aimed to adapt the e-health impact questionnaire, which was developed to allow comparison of two or more websites containing health information, into Turkish, and to conduct a reliability and validity study.

**Methods:**

This study is in methodological research design. The first part consists of items containing “general attitudes towards health-related websites”. This section consists of 11 items, two of which are sub-dimensions. The second part consists of items containing “opinions about the website reviewed”. This section contains a total of 26 items, three of which are sub-dimensions. SPPS and AMOS package programs were used in the analysis of the data.

**Results:**

It is seen that 18% of the individuals participating in the study (*n* = 388) were male and 82% were female. While 85.1% of them are studying or have studied at the undergraduate level, 14.9% of them are in postgraduate education. Part 1 of the questionnaire are as follows: (*χ*2/df) = 3.64 (*χ*2 = 156,843/df = 43); RMSEA = 0.08; GFI = 0.93; AGFI = 0.89; CFI = 0.92; TLI = 0.90; PGFI = 0.61 and NFI = 0.90. Part 2 are as follows: 4.81 (*χ*2/df) (*χ*2 = 1423.541/df = 296); RMSEA = 0.099; GFI = 0.75; AGFI = 0.70; CFI = 0.82; TLI = 0.80; PGFI = 0.63 and NFI = 0.79.

**Conclusion:**

This study suggests using this instrument to survey perceptions of e-health technology in Turkish people.

## Introduction

e-Health refers to the use of information and communication technologies (ICT) for health-related services. e-Health aims to distribute scarce resources to areas in need more efficiently and to reduce the cost of health care delivery. In addition, e-health provides timely and equal access to health services for all. The principle of Empowering Consumers and Patients, one of the basic principles of the e-Health concept, aims to provide health information to users by opening them to internet access (1). However, this information must be understandable by individuals. From an individual perspective, health literacy levels is expected to be high.

In the Health Literacy literature, literacy requires not only the individual's access to necessary health information, but also the understanding of the information obtained in order to increase health awareness and protect and improve health, and to use it in the right place at the right time (2). Studies in the literature state that most of the adult individuals have limited health literacy levels. This limitation often results in difficulties in understanding asymmetrical or highly complex health information and making informed health decisions. In addition, thanks to internet-connected technologies such as smart phones and computers, which are accessible to everyone today, individuals have faster access to information about their health. In this sense, the internet has quickly become one of the most popular sources of information. However, although the health information on the web-based internet is sometimes provided by specialist and similar health professionals, it should be stated that the opposite can also happen (3, 4). It is possible to state that individuals encounter various obstacles in order to correctly understand and apply the information they have obtained through websites, both due to the existing asymmetrical information and as a result of misdirection of individuals affected by similar diseases in forums and similar web-based environments. In order to help individuals overcome these obstacles and enable them to interact successfully with the content of web-based health services, sometimes their level of literacy and awareness needs to be increased, while sometimes it is seen that the problem is related to technical issues such as the language of the website and the visuals used. When examined in terms of these two situations, although the solution to the problem of obtaining information seems primarily to improve individual behaviors, technical issues such as improving the websites used for information purposes are also important. Unfortunately, there are no standardized criteria for evaluating health information on the Internet (5, 6).

In this study, it is aimed to adapt the e-health impact questionnaire, which was developed to allow comparison of two or more websites containing health information, into Turkish, and to conduct a reliability and validity study (7). The study was carried out on the basis of the principles determined and recommended by the International Test Commission (ITC). After the adaptation study is carried out, it is aimed to ensure that health professionals, researchers and web developers are informed about the experiences of using different types of materials (numbers, blogs, experiences, pictures, etc.) that users can add to their websites, while remaining true to the purpose of developing the original measurement tool (8–10).

## Materials and methods

### This study is in methodological research design

#### Original measuring tool

The original language of the e-Health Impact Questionnaire developed by Kelly et al. (7) is English and it is in two parts. The questionnaire is in a 5-point Likert type and ranges from Strongly disagree to Strongly agree (7).

Part 1 consists of items including “Attitudes towards online Health Information”. This part consists of 11 items, two of which are subscales. Items marked with * are reverse scored.

a1. Five questions (A1, A2, A3, A4, A5) about the first subscale “Attitudes towards online health information”;

a2. The second subscale “Attitudes towards sharing health experiences online” (A6, A7, A8, A9, A10, A11) consists of six questions.

Part 2 consists of items including “Attitudes towards Sharing Health Experiences Online”. This part contains a total of 26 items, three of which are subscales.

b1. The first subscale “Confidence and identification” (B14, B15, B19, B20 B18, B23, B17, B11, B10) consists of 9 questions;

b2. The second subscale “Information and presentation” (B26, B6, B9, B12, B3*, B5, B24, B25*) 8 questions;

b3. The third subscale “Understanding and motivation” (B1, B4, B22, B21, B7, B8, B16, B2, B13) includes 9 questions (7).

### Translation process

A six-stage path was followed for language validity (10–12). First, the Turkish translation of the original questionnaire was made by two English language experts. Secondly, a single form was obtained by comparing the questionnaire items translated by the researchers. In the third stage, the questionnaire items obtained in the second stage were translated back into English with two experts in the field of English language and two instructors in the field of Health Sciences. In the fourth stage, the questionnaire items that were translated back into English were analyzed by two independent researchers and turned into a single form. In the fifth stage, the form obtained in the fourth stage and the original questionnaire items were compared and the meaning changes, word and expression changes were compared and it was observed that there was no change. In the sixth and last stage, the draft Turkish version of the questionnaire was prepared. In order to determine the suitability for language and culture, a total of eight experts were consulted and pilot implementation was started.

In addition to the linguistic validation, cultural equivalence was ensured by consulting experts with deep knowledge of Turkish health communication culture. For instance, visual expressions and emotional vocabulary were adapted to align with Turkish health-seeking behavior, which may differ from the UK context where the original scale was developed.

### Pilot study

In order to understand the conformity of the translation of the questionnaire from its original language to Turkish, a pilot study was conducted with 20 participants who have advanced knowledge of both languages, and the conformity of the questions was evaluated.

### Population and sample

The population of the study consists of academicians of a university and students of Faculty of Health Sciences and Faculty of Nursing. The number of questionnaire items was taken into account in calculating the sample size. There are opinions that 5–10 times the number of items in the questionnaire can be selected (12). In this study, a total of 388 participants were reached by online methods, 10 times the number of questionnaire items (37 items in two parts). Although simple random sampling was initially considered, a convenience sampling method was ultimately used due to practical constraints. Participants were recruited voluntarily through online announcements shared with students and academic staff from the Faculty of Health Sciences and the Faculty of Nursing. The data of the study were collected between February and June 2022.

Although the study employed simple random sampling, the participants were drawn from health sciences students and academicians from a single university. Therefore, generalizability to the broader Turkish population may be limited. This limitation may affect the representativeness of e-health literacy and attitudes, especially among less educated or older populations.

### Analysis of data

SPSS 25 and AMOS 22 programs were used in the analysis of the data. Values such as percentage calculations and reliability coefficients were performed in the SPSS program, and Confirmatory Factor Analysis (CFA) was performed in the AMOS program. In the study, content and factor validity were used to examine the validity of the questionnaire. CFA was used for structural validity. Only CFA can be sufficient for questionnaire adaptation studies from another language to Turkish (13). The reliability of the questionnaire was tested with the Cronbach's Alpha reliability coefficient and the split-half method.

## Results

### Sociodemographic characteristics of the participants

The sociodemographic characteristics of the individuals participating in the study are shown in Table 1. It is seen that 18% of the individuals participating in the study (*n* = 388) were male and 82% were female. While 85.1% of them are studying or have studied at the undergraduate level, 14.9% of them are in postgraduate education. In addition, 13.4% of the participants are married and 86.6% are single.

**Table 1:** Sociodemographic characteristics of the participants.

Sociodemographic characteristics		
Age (*n*/mean/median)	467/27,39/27,00	
Gender	*n*	%
Male	70	18
Female	318	82
Total	388	100
Educational status	n	%
Undergraduate	330	85,1
Postgraduate	58	14,9
Total	388	100,0
Marital status	n	%
Married	53	13,4
Single	335	86,6
Total	388	100,0

*n* = number of participants.

The value 467 refers to the total number of records before data cleaning. After excluding incomplete responses, the final sample consisted of 388 participants, which was used for all subsequent analyses.

### Findings related to the validity of the questionnaire—validity

To verify the validity of the questionnaire, content validity and structural validity were examined.

### Content validity

In the study, ten expert opinions were taken to ensure the language, culture and content validity of the e-Health Impact Questionnaire items translated into Turkish (7). The items were arranged in line with the suggestions of the experts and it was found appropriate that the name of the questionnaire, which is “e-Health Impact”, should be “ e-Sağlık Etkisi (e-Health Impact)”.

### Structural validity

The item-total test correlation was calculated for the validity of the items (Table 2). Corrected item-total correlation coefficients for questionnaire items vary between 0.39 and 0.75, except for item 25 (−0.004) (14).

**Table 2:** Item-total test correlations for questionnaire items.

	Corrected Item-total correlation	Squared Multiple correlation	Cronbach's Alpha if Item deleted
A1	0.50	0.49	0.95
A2	0.53	0.50	0.94
A3	0.34	0.40	0.95
A4	0.34	0.48	0.95
A5	0.41	0.41	0.95
A6	0.50	0.52	0.95
A7	0.47	0.50	0.95
A8	0.48	0.40	0.95
A9	0.57	0.50	0.95
A10	0.58	0.57	0.95
A11	0.58	0.54	0.95
B1	0.67	0.56	0.95
B2	0.63	0.63	0.95
B3	0.63	0.30	0.95
B4	0.64	0.58	0.95
B5	0.50	0.43	0.95
B6	0.58	0.57	0.95
B7	0.63	0.55	0.95
B8	0.56	0.50	0.95
B9	0.51	0.53	0.95
B10	0.70	0.63	0.95
B11	0.68	0.62	0.95
B12	0.70	0.65	0.95
B13	0.56	0.52	0.95
B14	0.65	0.66	0.95
B15	0.72	0.70	0.95
B16	0.73	0.73	0.95
B17	0.75	0.72	0.95
B18	0.75	0.70	0.95
B19	0.71	0.65	0.95
B20	0.73	0.70	0.95
B21	0.72	0.65	0.95
B22	0.71	0.67	0.95
B23	0.71	0.67	0.95
B24	0.55	0.59	0.95
B25	0.004	0.33	0.95
B26	0.39	0.45	0.95

CFA was performed using the AMOS program to test the structural validity of the scale and to verify the compatibility of its subscales. In the original scale, a structure consisting of two domains in the first part and three domains in the second part was revealed as a result of the factor analysis performed on the sample consisting of men and women aged 18 and over who live in England and have access to the internet (6). In this study, since a structure with known factors was tested, the maximum likelihood technique was used in factor analysis

### CFA

The ratio of chi-square statistics to degrees of freedom obtained as a result of the analysis for Part 1 of the questionnaire are as follows: (*χ*2/df) = 3.64 (*χ*2 = 156,843/ df = 43); RMSEA = 0.08; GFI = 0.93; AGFI = 0.89; CFI = 0.92; TLI = 0.90; PGFI = 0.61 and NFI = 0.90. The compatibility values for Part 1 are acceptable (Table 3).

**Table 3:** Value ranges in the literature for AMOS analysis.

Index	Reference values good	Reference values acceptable value	Part 1	Part 2
*χ*2/sd	<2	<5	3.64	4.03
RMSEA	<0.05	<0.08	0.08	0.08
GFI	>0.90	>0.80	0.93	0.80
AGFI	>0.85	>0.75	0.89	0.76
CFI	>0.95	>0.90	0.92	0.87
TLI	>0.95	>0.90	0.90	0.85
NFI	>0.95	>0.90	0.90	0.84
Pgfi	>0.50		0.61	0.66

Source (16–25).

**Table 4:** Split-half analysis.

Part 1		Single questions	Double questions
Single questions	r	1	0.79*
Double questions	r	0.79*	1
Part 2		Single questions	Double questions
Single questions	r	1	0.94*
Double questions	r	0.94*	1

*n* = 388.

* *p* < 0,01 *p* = 0.00.

As a result of CFA, the ratio of chi-square statistics to degrees of freedom for Part 2 are as follows: 4.81 (*χ*2/df) (*χ*2 = 1,423.541/df = 296); RMSEA = 0.099; GFI = 0.75; AGFI = 0.70; CFI = 0.82; TLI = 0.80; PGFI = 0.63 and NFI = 0.79. Looking at the results, it is seen that the fit indices are not at an acceptable level. In addition, the factor load of the 25th item in b2 “*I found the images on the website distressing*”, which is one of the subscale of the part, was removed from the questionnaire because it was 0.21 (<30). Since a structure whose factors are known before is tested in the created model, some questionnaire items need to be removed from the questionnaire until the test model reaches the fit values (15). Therefore, CFA was applied to the questionnaire again over a total of 25 items. In the light of the findings obtained, modifications were made between the 6th and 7th, 8th and 9th, 5th and 20th, 14th and 20th items of Part 2 in order to ensure that the goodness of fit indices were at the desired level. After the modification, this part was reanalyzed with the remaining 25 items. The model created in CFA is presented in Table 3.

The lower fit indices observed in Part 2 (especially CFI = 0.87, TLI = 0.85) might be attributed to cultural differences in interpreting certain items related to personal health experience sharing. Turkish participants may perceive some items (e.g., B25 on distressing visuals) differently due to cultural norms around emotional expression and trust in health content, affecting factor loadings.

As a result of the final CFA, the ratio of chi-square statistics to degrees of freedom for Part was found as 2 (*χ*2/df) = 4.03 (*χ*2 = 1,081,220/df = 268). This value is expected to be ≤5 (16). Other fit indices were calculated as RMSEA = 0.08; GFI = 0.80; AGFI = 0.76; CFI = 0.87; TLI = 0.85; PGFI = 0.66 and NFI = 0.84. Factors such as the size of the sample size and the change in correlation seem to affect indices such as CFI and TLI. Considering that the RMSEA value for both parts is in the good fit range, the reason for the low CFI and TLI values can be explained in this way (26). The fit values for Part 2 are also at an acceptable level (Table 3).

**Figure 1:**
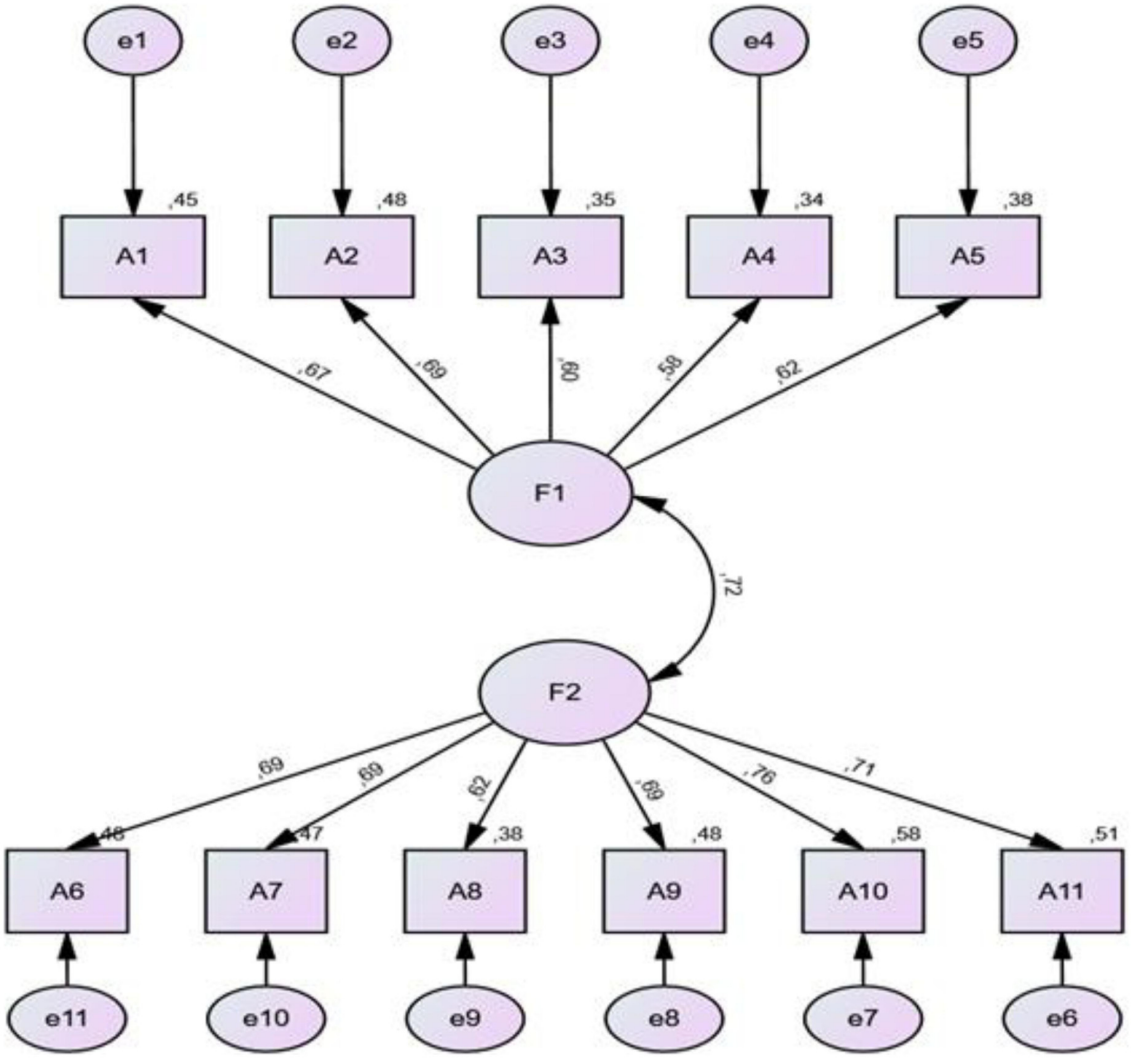
CFA results for the part on general attitudes towards health-related websites (part 1). When the factor loads of part 2 are examined.

The factor loads obtained as a result of the CFA performed on the items of the “general attitudes towards health-related websites” in Chapter 1, ranged between 0.58 and 0.69 for the “attitude towards online health information (a1)” subscale, and 0.62 and 0.76 for the “sharing of online health experience (a2)” subscale (Figure 1).

**Figure 2:**
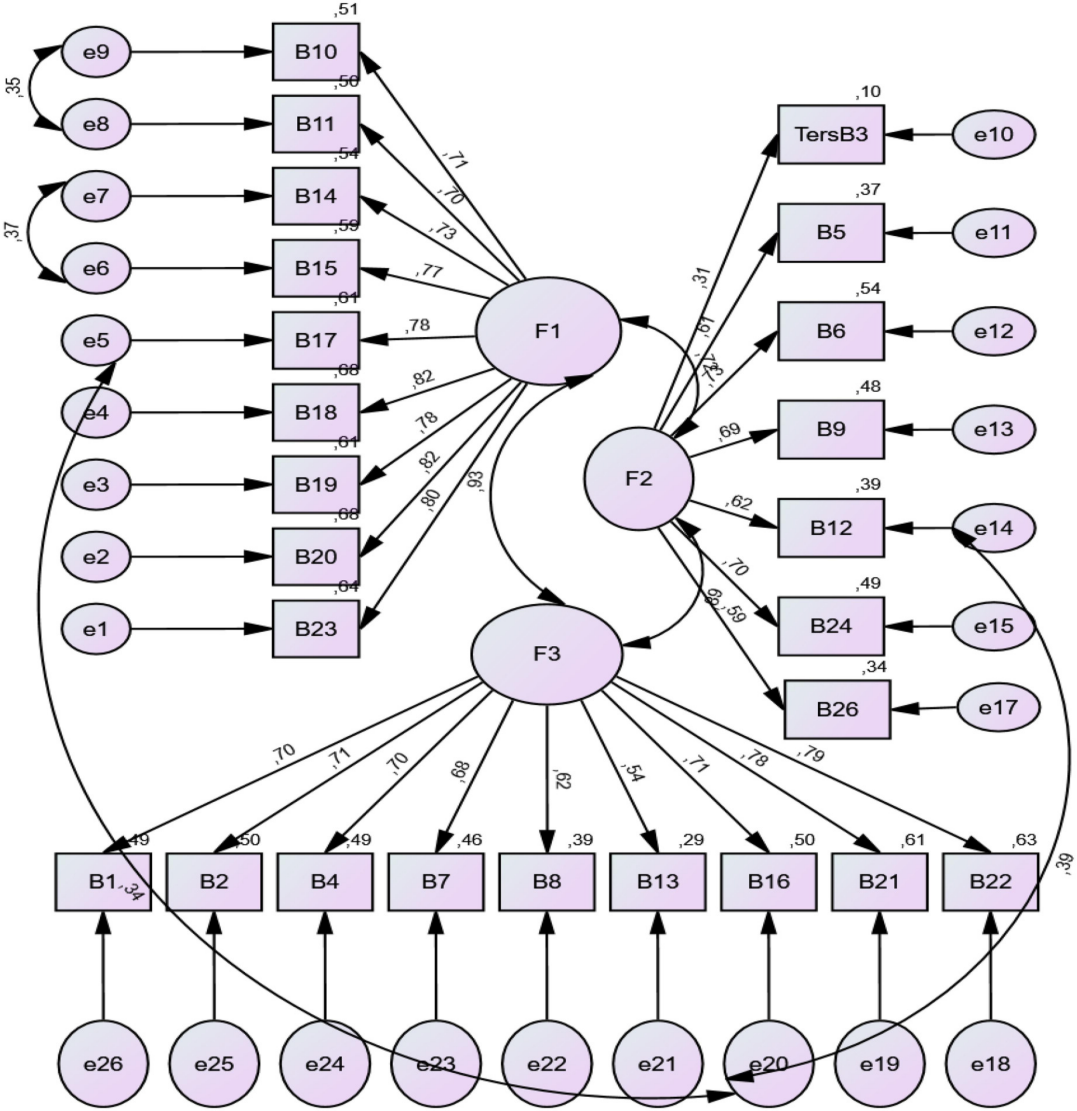
CFA results of the opinions about the reviewed website (part 2).

The factor loads of Part 2 were determined between 0.70 and 0.82 for “Confidence and identification (b1)” subscale, 0.31 and 0.73 for “Information and presentation (b2)” subscale and 0.54 and 0.79 for “Understanding and motivation (b3)” subscale. The fact that the factor loads obtained are greater than 0.30 means that the factor loads of the items are sufficient (27). The goodness of fit in the CFA for the structural validity of the scale is acceptable for both parts (Figures 1,2).

## Model fitness of confirmatory factor analysis model

### Findings related to the reliability of the questionnaire-reliability

#### Internal consistency of the scale

Cronbach Alpha reliability coefficient and Split-Half Reliability were used to determine the internal consistency reliability of the scale.

#### Cronbach alpha reliability coefficient

In the study, the Cronbach Alpha value of the total items for Part 1 was calculated as 0.87. In the subscale of Part 1, values of 0.77 for a1 and 0.84 for a2 were obtained. The Cronbach Alpha value of the total items for Part 2 of the scale was found to be 0.95. The Cronbach Alpha coefficients of the subscales were found to be b1 = 0.93; b2 = 0.78 and b3 = 0.89. The acceptability level of this value is specified as 0.70 and above (28, 29). It is seen that the Cronbach Alpha value of the scale is high. In addition, it was observed that the Cronbach's alpha if item deleted value of the scale varied between 0.946 and 0.949 (Table 2).

#### Split-half method

The internal consistency of the two-part scale was determined by split-half method. The correlation coefficient between the two halves of the scale was 0.79 in Part 1 and 0.94 in Part 2 (*p* < 0.01) (Table 4). Since this coefficient was above 0.70, the internal consistency of the test was evaluated as high (13).

The descriptive findings of the participants are presented in Table 5.

**Table 5:** Descriptive findings of the participants.

	Min	Max	Mean	Std. Dev.
A	1.09	5.00	3.18	0.73
A1	1.00	5.00	2.83	0.85
A2	1.00	5.00	3.47	0.81
B	1.20	5.00	3.32	0.71
B1	1.00	5.00	3.08	0.87
B2	1.14	5.00	3.66	0.66
B3	1.00	5.00	3.32	0.76

*n* = 388

When the descriptive findings are examined, it is seen that the overall mean for Part 1 is 3.18 ± 0.73 and for Part 2 it is 3.32 ± 0.71. Except for the a1 and b1 subscale, the other domains (mean value = 3) were above the middle (Table 5).

## Discussion

In the study, it was aimed to adapt the “e-Health Impact” questionnaire developed by Kelly at al. (7) into Turkish. In the original questionnaire, the study was conducted with 221 participants over the age of 18 having internet access (6). The study was carried out with 388 participants, including academicians working at a university and students from the Faculty of Health Sciences and the Faculty of Nursing.

According to the descriptive statistics of the participants, the average of “attitudes towards online health information (a1)” in Part 1 “general attitudes towards health-related websites” was lower than the other subscales; this may be due to the negative attitudes of the participants towards online health information. It is also observed that the “Confidence and identification (b1)” subscale in the “ opinions about the reviewed website” part is also lower than the average of the second part. Therefore, it can be concluded that the attitudes and confidence of the participants towards online health information are low.

In order to test the validity of the scale, the structural validity was checked. When the item-total score correlation coefficients of the scale were examined, it was observed that the other items except one item had high values. For structural validity, the parts of the scale and the factor structure, which has two domains in the first part and three domains in the second part, were tried to be verified with the maximum likelihood estimation method. As a result of the CFA, it was determined that the fit indices of Part 1 were at an acceptable level. As a result of the CFA performed in Part 2 of the questionnaire, it was observed that the fit indexes of the three-factor structure were lower than the acceptable values. Since a structure with known domains was tested before, the item number 25 of the second part, “I found the images on the website distressing”, was removed from the questionnaire, taking into account the item-total correlations with the items of the questionnaire with a low factor load. Item 25 removed from the questionnaire was in the “information and presentation (b2)” subscale of the “general opinions about the examined website” part. After the last item removed, CFA was applied to the remaining questionnaire with 25 evaluation items and in the light of the findings obtained, in order to ensure that the goodness of fit indices were at the desired level, articles 6 and 7, 8 and 9, 5 and 20, 14 and 20 of this section have been modified. After the modification, the remaining 25 items and Part 2 were analyzed again and sufficient fit values were reached. Thus, the three-factor structure, which overlaps with the original questionnaire and includes the domains of “confidence and identification (b1)”, “knowledge and presentation (b2)” and “understanding and motivation (b3)”, was confirmed. It was determined that the fit indices of the three-factor structure of the second part were among acceptable values.

Particularly, the low factor loading for Item 25 and moderate loadings for some items in the “Information and presentation” subscale may indicate cultural discomfort or ambiguity around visual elements on health websites. Turkish users may focus more on textual rather than visual content, leading to lower consistency in responses.

For the reliability of the e-Health Impact questionnaire, its internal consistency was checked. Cronbach's Alpha coefficient and split-half analyzes were performed to determine internal consistency. The Cronbach Alpha values obtained for the parts and subscales of the questionnaire were found to be high. The values obtained show parallelism with the values of the questionnaire in previous studies (7). Since the correlation coefficient between the two halves of the questionnaire was above 0.70, the internal consistency of the test was evaluated as high.

This study offers a crucial step toward enabling structured evaluation of online health information tools in the Turkish language. As the digitalization of health services accelerates, having a culturally adapted and psychometrically sound instrument to measure e-health impact is essential for improving public health communication and accessibility.

## Conclusion and recommendations

As a result, a measurement tool that can measure the clarity of the information on a web-based site about health, the suitability of pictures and figures, or the effects of the ideas shared by individuals on web-based platforms, has been adapted into Turkish, ensuring its validity and reliability. In its final form, the questionnaire consists of two parts. Part 1 consists of two subscales and Psrt 2 consists of three subscales. The final version of the questionnaire is in Supplementary Appendix 1.

## Data Availability

The raw data supporting the conclusions of this article will be made available by the authors, without undue reservation.
