# Supplementary material for: Adaptation of e-health impact questionnaire into Turkish: a validity and reliability study
Source: Front Digit Health. 2025 Sep 30;7:1538475. doi: 10.3389/fdgth.2025.1538475 (PMC12518320; doi:10.3389/fdgth.2025.1538475)
Supplement: Supplementary file 1 [file Datasheet1.pdf]

SUPPLEMENTARY MATERIAL. Turkish Version Of The Scale

BÖLÜM 1

Bu bölüm, web siteleri hakkında genel görüşlerinizi sormaktadır.

	Uygun olan kutucuğu işaretleyiniz.				
**Aşağıdaki ifadelere ne ölçüde katıldığınızı belirtiniz**	**Kesinlikle Katılmıyorum**	**Katılmıyorum**	**Ne katılıyorum Ne katılmıyorum**	**Katılıyorum**	**Kesinlikli Katılıyorum**
1. İnternet, doktorun bana söylediklerini anlamama yardımcı olacak güvenilir bir kaynaktır.					
2. İnternet, insanların bir sağlık sorunuyla yaşamanın nasıl bir şey olduğunu bilmelerine yardımcı olabilir.					
3. İnternet, insanların semptomlarının doktora gidecek kadar önemli olup olmadığına karar vermelerine yardımcı olmak için yararlı olabilir.					
4. Sağlığımla ilgili bir karar vermek için yardıma ihtiyacım olursa interneti kullanırdım (örneğin, bir doktora muayene olmam, ilaç almam veya başka tedaviler aramam gerekip gerekmediği).					
5. Doktorun bana uygun tavsiyede bulunup bulunmadığını kontrol etmek için interneti kullanırdım.					
6. İnternet, benzer sağlık sorunları yaşayan diğer insanları bulmanın iyi bir yoludur.					
7. İnternet, diğer insanların sağlıkla ilgili deneyimlerini okumak faydalı olabilir.					
8. Çevrenizdeki insanlara (örneğin, aileniz veya işteki insanlar) nasıl hissettiğinizi söylemek istemiyorsanız, internet yararlıdır.					
9. Sağlıkla ilgili web sitelerine günün veya gecenin herhangi bir saatinde erişebileceğimi bilmek güven verici olabilir.					
10. İnternet, karşı karşıya kalabileceğim sağlık ile ilgili kararlarla karşı karşıya kalan diğer insanları					

bulmanın iyi bir yoludur.					
11. Sağlık web sitelerine bakmak, sağlık endişelerimde yalnız olmadığıma dair bana güven veriyor.					

Lütfen aşağıdaki talimatları izleyin:

1. Aşağıdaki sağlıkla ilgili web sitesinin bağlantısını tıklayın. Bu, tarayıcınızda yeni bir sayfa açacaktır.)
2. Bu web sitesinin ilginizi çeken alanlarına göz atmak için lütfen 10-15 dakikanızı ayırın.)
3. Web sitesine göz atmayı tamamladığınızda, bu sayfaya geri dönün ve kalan soruları tamamlamak için 'devam et'i tıklayın.)

(https://www.incelenecek web sayfası.com/)

(Bu anket için 30 dakikanız olduğunu ve bu süre içinde tamamlanmazsa geçersiz olacağını lütfen unutmayın)

BÖLÜM 2

Bu bölüm, az önce baktığınız sağlık ile ilgili web sitesi hakkındaki görüşlerinizi sormaktadır.

	Uygun olan kutucuğu işaretleyiniz.				
Az önce incelediğiniz web sitesini düşündüğünüzde, aşağıdaki ifadelere ne ölçüde katılıyorsunuz veya katılmıyorsunuz?	**Kesinlikle Katılmıyorum**	**Katılmıyorum**	**Ne katılıyorum Ne katılmıyorum**	**Katılıyorum**	**Kesinlikli Katılıyorum**
1. Web sitesi, sağlığım için faydalı olabilecek eylemlerde bulunmam için beni teşvik ediyor.					
1. Web sitesi olumlu bir görünüme sahiptir.					
2. Web sitesindeki bilgiler kafamı karıştırdı.					
3. Web sitesi, hayatı nasıl daha iyi hale getireceğinize dair faydalı ipuçları içerir.					
4. Web sitesi geniş bir bilgi yelpazesi sunar.					
5. Web sitesindeki dil anlaşılmasını kolaylaştırdı.					

6. Web sitesini ziyaret ettikten sonra kendime bakmaya daha meyilli hissediyorum.					
7. Siteden yeni bir şey öğrendim.					
8. Web sitesindeki bilgileri kolayca anlayabiliyorum.					
9. Web sitesi benim sağlığıma ne olabileceğine hazırlıyor.					
10. Web sitesine katkıda bulunan insanlar benim için neyin önemli olduğunu anlıyor.					
11. Web sitesindeki bilgilere güveniyorum.					
12. Sağlığımla ilgili bir karar vermem gerekirse web sitesine danışırdım.					
13. Web sitesini kullanan diğer insanlarla dayanışma duygusuna sahip olduğumu hissediyorum.					
14. Web sitesini kullanan diğer insanlarla ortaklaklık hissedebilirim.					
15. Genel olarak, web sitesini güven verici buluyorum.					
16. Web sitesinde verilen tavsiyelere değer veriyorum.					
17. Web sitesi bana sağlığımı yönetebileceğime dair güven veriyor.					
18. Web sitesini kullanan diğer insanlarla çok ortak noktam olduğunu hissediyorum.					
19. Web sitesi, sağlıkla ilgili endişelerimi başkalarına açıklamam için bana güven veriyor.					
20. Web sitesi kişisel sağlığımı daha iyi anlamama yardımcı oluyor.					
21. Web sitesi beni sağlık bakımımda daha aktif bir rol oynamaya teşvik ediyor.					
22. Web sitesi, sağlığımı çevremdeki insanlarla tartışma konusunda kendime daha fazla güvenmemi sağlıyor (örneğin, ailem veya işteki kişiler).					
23. Web sitesinde fotoğraflar ve diğer görseller uygun şekilde kullanılmıştır.					

24. Web sitesinin kullanımı kolaydır.					
